# A phase I study of the safety and efficacy of talimogene laherparepvec in Japanese patients with advanced melanoma

**DOI:** 10.1111/cas.15450

**Published:** 2022-06-30

**Authors:** Naoya Yamazaki, Taiki Isei, Yoshio Kiyohara, Hiroshi Koga, Takashi Kojima, Tatsuya Takenouchi, Kenji Yokota, Kenjiro Namikawa, Min Yi, Alissa Keegan, Satoshi Fukushima

**Affiliations:** ^1^ Department of Dermatologic Oncology National Cancer Center Hospital Tokyo Japan; ^2^ Department of Dermatologic Oncology Osaka International Cancer Institute Osaka Japan; ^3^ Division of Dermatology Shizuoka Cancer Center Hospital Shizuoka Japan; ^4^ Department of Dermatology Shinshu University School of Medicine Matsumoto, Nagano Japan; ^5^ Department of Gastroenterology and Gastrointestinal Oncology National Cancer Center Hospital East Kashiwa‐shi, Chiba Japan; ^6^ Division of Dermatology Niigata Cancer Center Hospital Niigata‐shi, Niigata Japan; ^7^ Department of Dermatology Nagoya University Hospital Nagoya‐shi, Aichi Japan; ^8^ Amgen Inc. Thousand Oaks California USA; ^9^ Department of Dermatology and Plastic Surgery Faculty of Life Sciences Kumamoto University Kumamoto Japan

**Keywords:** immunotherapy, Japanese, melanoma, phase I clinical trial, talimogene laherparepvec

## Abstract

Talimogene laherparepvec (T‐VEC) is approved for the treatment of unresectable melanoma in the USA, Europe, and Australia. This phase I, multicenter, open‐label, dose de‐escalation study evaluated the safety and efficacy of T‐VEC in Japanese patients with unresectable stage IIIB–IV melanoma. Eligible adult patients had histologically confirmed stage IIIB–IVM1c cutaneous melanoma, may have received prior systemic anticancer therapy, must have had ≥1 injectable lesion, serum lactate dehydrogenase ≤1.5x upper limit of normal, ECOG performance status of 0 or 1, and adequate hematologic, hepatic, and renal function. T‐VEC was injected intralesionally (first dose, ≤4.0 ml of 10^6^ PFU/ml; after 3 weeks and then every 2 weeks thereafter, ≤4.0 ml of 10^8^ PFU/ml). Primary endpoints were dose‐limiting toxicities (DLTs) and durable response rate (DRR). Of 18 enrolled patients (72.2% female), 16 had received ≥1 prior line of therapy. Ten patients discontinued T‐VEC due to disease progression. Median (range) follow‐up was 20.0 (4–37) months. No DLTs were observed; 17 (94.4%) patients had treatment‐emergent adverse events (AEs). Fourteen (77.8%) patients had treatment‐related AEs; the most frequent were pyrexia (44.4%), malaise (16.7%), chills, decreased appetite, pruritus, and skin ulcer (11.1% each). The primary efficacy endpoint was met: 2 (11.1%) patients had a durable partial response ≥6 months. The DRR was consistent with that observed in a phase III trial of T‐VEC in non‐Asian patients. The safety profile was consistent with the patients' underlying disease and the known safety profile of T‐VEC.

## Graphical abstract

This phase I study assessed the safety and efficacy of talimogene laherparepvec (T‐VEC) in Japanese patients with advanced melanoma including rare subtypes. The safety profile was similar to previous reports of T‐VEC monotherapy, and the durable response rate was consistent with that observed in non‐Asian patients.

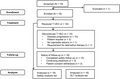

**Abbreviations**
AE: adverse event
ALM: acral lentiginous melanoma
CI: confidence interval
CR: complete response
DLT: dose‐limiting toxicity
DOR: duration of response
DRR: durable response rate
FDA: food and drug administration
GM‐CSF: granulocyte‐macrophage colony‐stimulating factor
HSV‐1: herpes simplex virus 1
LMM: lentigo maligna melanoma
MM: mucosal melanoma
NE: not estimable
NM: nodular melanoma
OPTiM: Oncovex(GM‐CSF) Pivotal Trial in Melanoma
ORR: objective response rate
OS: overall survival
PFS: progression‐free survival
PR: partial response
SAE: serious adverse event
SD: standard deviation
SSM: superficial spreading melanoma
TEAE: treatment‐emergent adverse event
TRAE: treatment‐related adverse event
TTR: time to response
T‐VEC: talimogene laherparepvec
WHO: World Health Organization

## 1 INTRODUCTION

Melanoma is an aggressive skin cancer, with an increasing incidence worldwide.^1^, ^2^ It is the third most common type of skin cancer in Japan and accounts for ~50% of the mortality from skin cancers.^3^ Melanoma has been classified into clinicopathologically distinguishable subtypes such as cutaneous, mucosal, uveal, and unknown primary melanomas.^3^ Cutaneous melanomas are further categorized into SSM, NM, LMM, and ALM.^4^ Although less frequent overall, the incidence of ALM and MM is higher in the Japanese population than in non‐Asian populations.^5^, ^6^, ^7^

While early‐stage melanoma is usually curatively treated with surgery, melanoma with distant metastases is rarely resectable.^8^ The prognosis for metastatic melanoma is poor, with a median OS of 6.2 months.^9^ The 5‐year survival rates vary widely depending on the disease stage, ranging from 99% for localized melanoma to 66% and 27% for melanoma with regional and distant metastases, respectively.^2^ In Japan, the 10‐year OS rates for stage III and stage IV disease were reported as 54% and 7%, respectively.^10^

The application of checkpoint inhibitors (ipilimumab, nivolumab, pembrolizumab) and small molecule inhibitors of BRAF and MEK (vemurafenib, dabrafenib, trametinib, cobimetinib, binimetinib, encorafenib) has transformed the outcomes for patients with advanced melanoma.^3^, ^11^ According to the 2019 Japanese Dermatological Association guidelines, the current treatment regimen for Japanese patients with melanoma comprises dabrafenib plus trametinib, encorafenib plus binimetinib, pembrolizumab monotherapy, and nivolumab alone or in combination with ipilimumab.^3^, ^12^ Vemurafenib (December 2014), dabrafenib plus trametinib (March 2016), and encorafenib plus binimetinib (January 2019) were approved in Japan following trials that demonstrated their efficacy and safety in melanoma patients.^13^, ^14^, ^15^, ^16^, ^17^ Ipilimumab showed acceptable tolerability in a phase II trial in Japanese patients with advanced melanoma, with best ORR and disease control rate of 10% and 20%, respectively.^18^ Phase II trials of nivolumab have been conducted in previously untreated and treated Japanese patients with advanced melanoma; the ORR and median OS in these trials were 35% and 33 months, and 29% and 18 months, respectively.^19^, ^20^, ^21^, ^22^ Moreover, a phase Ib trial of pembrolizumab in patients with cutaneous melanoma demonstrated a confirmed ORR of 24%.^23^

Despite these recent approvals, additional treatment options are needed to further improve outcomes with minimal toxicity in patients with metastatic melanoma in Japan. Vemurafenib, dabrafenib, and trametinib are associated with early development of resistance in most cases, leading to short DORs. Furthermore, these treatments are associated with severe and long‐lasting toxicities. T‐VEC is an intralesional oncolytic viral immunotherapy designed to produce GM‐CSF in tumors to enhance antigen release, presentation, and antitumor immune responses.^24^, ^25^ T‐VEC is the first FDA‐approved oncolytic viral therapy for the treatment of unresectable cutaneous, subcutaneous, and nodal lesions in patients with melanoma recurrent after initial surgery.^26^ The FDA approval was based on data from the OPTiM trial (stage IIIB–IVM1c melanoma), which showed that T‐VEC significantly improved the DRR versus subcutaneous GM‐CSF (16.3% vs 2.1%; *p* < 0.001).^27^ This phase I study was designed to evaluate the safety and efficacy of T‐VEC in Japanese patients with metastatic melanoma.

## 2 MATERIAL AND METHODS

### 2.1 Patients

Eligible patients (≥18 years) had histologically confirmed stage IIIB–IVM1c melanoma, could be treatment naïve, may have received prior systemic anticancer therapy, must have had ≥1 injectable cutaneous, subcutaneous, or nodal melanoma lesion ≥10 mm in longest diameter or multiple injectable melanoma lesions that in aggregate have a longest diameter of ≥10 mm, serum lactate dehydrogenase ≤1.5× upper limit of normal, ECOG performance status of 0–1, and adequate hematologic, hepatic, and renal function. Patients were excluded if they had previously received T‐VEC; active brain metastases, >3 visceral metastases (not including lung metastases or any nodal metastases associated with visceral organs), or any bone metastases; primary ocular or MM; history of symptomatic autoimmune disease or autoimmune disease that required systemic treatment; evidence of clinically significant immunosuppression; acute or chronic active hepatitis B or C infection or human immunodeficiency virus infection; active herpetic skin lesions; prior complications from herpetic infection; or required systemic antiherpetic treatment other than intermittent topical use.

### 2.2 Study design

This was a phase I, multicenter, open‐label, dose de‐escalation study to evaluate the safety and efficacy of T‐VEC monotherapy given at standard doses in Japanese patients with unresectable stage IIIB–IV melanoma (NCT03064763). Patients were enrolled from eight centers in Japan between March 2017 and March 2019 (Figure S1). The DLT evaluation period was 35 days from the initial administration of T‐VEC. All patients completed a safety follow‐up visit 30 (+7) days after the last dose of T‐VEC. Patients were followed for survival and T‐VEC–related AEs every 12 weeks (±28 days) for 24 months after the last patient was enrolled. For patients treated beyond 24 months after the last patient was enrolled, their final visit served as the safety follow‐up visit.

### 2.3 Dosage and treatment

Patients received T‐VEC (≤4.0 ml of 10^6^ PFU/ml [dose 1]) on day 1 of week 0 followed by ≤4.0 ml of 10^8^ PFU/ml after 3 weeks and every 2 weeks thereafter. If required, dose de‐escalation was permitted based on DLT evaluation of the first six DLT‐evaluable patients. T‐VEC was administered by intralesional injection into cutaneous, subcutaneous, and nodal tumors, with or without image ultrasound guidance. T‐VEC administration continued until the patient achieved a CR, had a DLT during the DLT evaluation period, had no injectable lesions, had clinically relevant disease progression beyond 24 weeks of treatment per modified WHO response criteria, had a safety concern, or had a maximum treatment duration of 48 months, whichever occurred first.

### 2.4 DLT evaluation

Dose‐limiting toxicities were defined as any of the following treatment‐related toxicities occurring during the DLT evaluation period: grade 4 nonhematologic toxicity; grade 3 nonhematologic toxicity lasting >3 days despite optimal supportive care (except grade 3 fatigue); grade ≥3 nonhematologic laboratory value requiring medical intervention/hospitalization or abnormality persisting >1 week; grade 3/4 febrile neutropenia; thrombocytopenia <25 × 10^9^/L associated with a bleeding event requiring intervention; serious herpetic event; grade 5 toxicity; or any toxicity requiring permanent discontinuation of T‐VEC.

Initially, six DLT‐evaluable patients were enrolled and treated with dose 1. The incidence of DLTs in the first six DLT‐evaluable patients and additional safety data from all patients were evaluated by a dose‐level review team comprising investigators and representatives of Amgen study teams. The dose level would be declared tolerable if the incidence of DLTs was <33% during the DLT evaluation period.

### 2.5 Endpoints and assessments

The primary endpoints were the incidence of DLTs and DRR using modified WHO response criteria.^28^ DRR was defined as the rate of CR or PR lasting continuously for ≥6 months and onset within 1 year of treatment. Secondary endpoints included ORR, TTR, DOR, and PFS using modified WHO response criteria, and OS.

Safety endpoints, in addition to DLTs, included the patient incidence of the following: all TEAEs, grade ≥3 TEAEs, SAEs, clinically relevant laboratory changes, and changes in vital signs. TEAEs were defined as AEs with an onset from the first dose of T‐VEC up to 30 days after the last dose of T‐VEC. SAEs were defined as AEs with an onset from the first dose of T‐VEC up to 90 days after the last dose of T‐VEC or 30 days following cessation of treatment if the patient initiated new anticancer therapy, whichever was earlier. TEAEs were coded using Medical Dictionary for Regulatory Activities and graded for severity using the US National Cancer Institute (NCI) Common Terminology Criteria for AE (v4.0). Fatal AEs and AEs leading to withdrawal of T‐VEC were also summarized.

### 2.6 Statistical analysis

Approximately 18 patients were to be enrolled and included in the safety and efficacy evaluations. The probability of declaring a dose level safe (unsafe) based on the six DLT‐evaluable patients was 89% (11%), 42% (58%), and 11% (89%) if the true DLT rate was 10%, 30%, or 50%, respectively.

A one‐sided 5% significance level exact binomial test was performed to test the null hypothesis of a 2% DRR. The expectation was that this test would be based on the first 18 patients who received ≥1 dose of T‐VEC. The null hypothesis was to be rejected if ≥2 patients achieved a durable response. Assuming a DRR of 16.3% consistent with that in the OPTiM study,^27^ this analysis had 81% power. DRR was summarized with an associated exact 95% CI. DOR among responders, TTR, PFS, and OS were estimated using the Kaplan–Meier method. ORR was summarized with an associated exact 95% CI.

## 3 RESULTS

### 3.1 Patients

**FIGURE 1:**
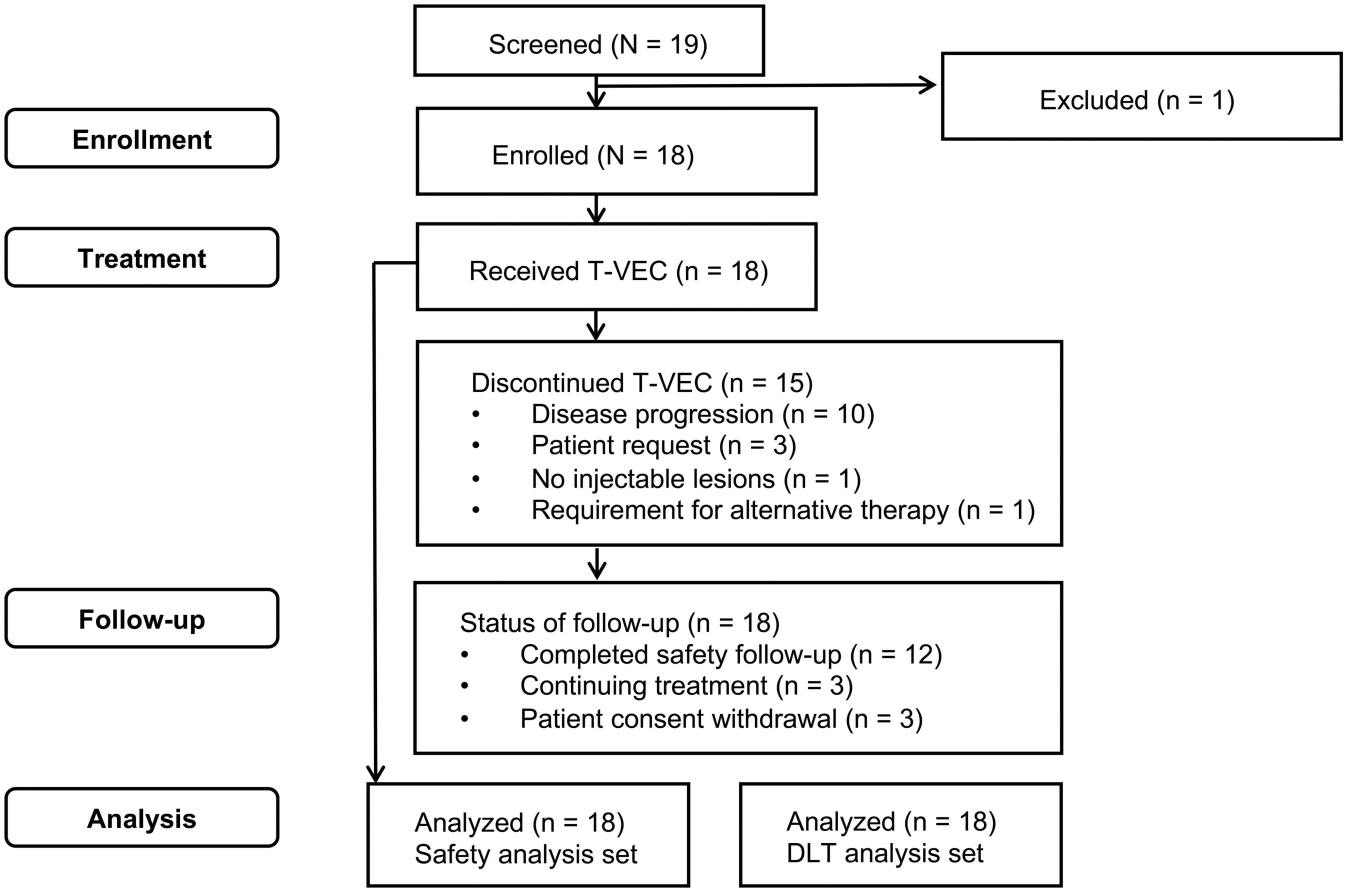
Patient disposition. DLT, dose‐limiting toxicity; qPCR, quantitative polymerase chain reaction; T‐VEC, talimogene laherparepvec. No patients (*n* = 0) were included in the qPCR analysis set. The DLT analysis set included all enrolled patients who were followed for ≥35 days on treatment from initial dosing (unless discontinued due to DLT) and had received ≥1 dose of T‐VEC, and was used to summarize the patient incidence of DLTs for the study. The safety analysis set included all enrolled patients who received ≥1 dose of T‐VEC and was used for analyzing safety endpoints other than patient incidence of DLTs. The qPCR analysis set included patients in the safety analysis set with a swab sample obtained for qPCR testing of T‐VEC DNA from any lesion suspected to be of herpetic origin

In total, 18 patients were enrolled, all of whom received ≥1 dose of T‐VEC (Figure 1). The number of lesions injected, lesion type injected, and the body site location injected for each patient for the first dose are provided in Table S1. Fifteen (83.3%) patients discontinued T‐VEC during the study due to disease progression (*n* = 10), patient request (*n* = 3), protocol‐specified criteria (*n* = 1; no injectable lesions), and requirement for alternative therapy (*n* = 1). At the time of the analysis, 3 (16.7%) patients were still receiving T‐VEC. Eight (44.4%) patients died during the course of the study and 10 (55.6%) patients were still on study at the time of the analysis. The median (range) follow‐up was 20.0 (4–37) months. Five (27.8%) patients had ≥1 important protocol deviation during the study.

Most patients (median age, 55 years) enrolled were female (72.2%) (Table 1). In total, 16 (88.9%) and 2 (11.1%) patients had an ECOG performance status of 0 and 1, respectively. Ten (55.6%) patients had stage IIIB–IVM1a disease, and 8 (44.4%) patients had stage IVM1b‐c disease (Table S1). The most frequent subtypes of melanoma were SSM and NM (27.8% each) followed by ALM (16.7%) and LMM (5.6%). The *BRAF* V600 mutation was observed in 22.2% of the patients. Eleven (61.1%) patients were HSV‐1 positive at baseline. All but two patients had received prior lines of therapy, including first‐line (16.7%), second‐line (38.9%), third‐line (27.8%), and >fourth‐line (5.6%). The most frequently reported types of prior anticancer therapy were immunotherapy (77.8%) and chemotherapy (11.1%), followed by radiotherapy (5.6%) (Table 1; Table S2).

**TABLE 1:** Baseline demographics and disease characteristics (safety analysis set; *n* = 18)

	T‐VEC (*n* = 18)
Sex	
Male	5 (27.8)
Female	13 (72.2)
Race	
Asian	18 (100.0)
Age, median (range), years	55 (28–83)
ECOG performance status^a^	
0	16 (88.9)
1	2 (11.1)
Histologic subtype	
Superficial spreading	5 (27.8)
Lentigo maligna	1 (5.6)
Acral lentiginous	3 (16.7)
Nodular	5 (27.8)
Desmoplastic	0 (0.0)
Unclassifiable	4 (22.2)
Disease stage (current)	
Stage IIIB	1 (5.6)
Stage IIIC	3 (16.7)
Stage IVM1a	6 (33.3)
Stage IVM1b	2 (11.1)
Stage IVM1c	6 (33.3)
*BRAF* status	
No mutation detected (WT *BRAF*)	12 (66.7)
*BRAF* V600K	0 (0.0)
*BRAF* V600E	4 (22.2)
Other *BRAF* mutation	0 (0.0)
Missing/unknown	2 (11.1)
Positive baseline HSV‐1 status	11 (61.1)
Prior therapies^b^	
Surgery	18 (100.0)
Radiotherapy	1 (5.6)
PD‐1/PD‐L1 therapy	11 (61.1)
Baseline LDH > ULN	9 (50.0)

*Note*: Data are presented as number (%) of patients, unless otherwise indicated. The safety analysis set included all enrolled patients who received ≥1 dose of T‐VEC.

Abbreviations: ECOG, Eastern Cooperative Oncology Group; HSV‐1, herpes simplex virus 1; LDH, lactate dehydrogenase; *n*, number of patients in the safety analysis set; PD‐1, programmed cell death protein 1; PD‐L1, programmed death‐ligand 1; T‐VEC, talimogene laherparepvec; ULN, upper limit of normal.

^a^ ECOG performance status: 0 = fully active, able to carry on all pre‐disease performance without restriction; 1 = restricted in physically strenuous activity but ambulatory and able to carry out work of a light or sedentary nature, for example, light housework, office work; only patients with ECOG 0 or 1 at baseline were allowed to be enrolled.

^b^ A patient could be counted more than once in this summary.

### 3.2 DLT

No patient had a DLT during the DLT evaluation period, and dose 1 was deemed tolerable. At the time of data cut‐off (August 3, 2020), the median (range) duration of treatment was 23.1 (3.1–89.1) weeks. The mean (SD) volume of T‐VEC was 2.77 (1.19) ml at first injection (10^6^ PFU/ml) and 2.80 (1.19) ml after the first injection (10^8^ PFU/ml), and the mean (SD) cumulative volume administered was 44.16 (40.07) ml.

### 3.3 Safety

Seventeen (94.4%) patients had ≥1 TEAE. The most frequently reported TEAEs (≥20% of patients) were pyrexia (50.0%), nasopharyngitis (27.8%), and malaise (22.2%) (Table 2). Grade 3 TEAEs occurred in 5 (27.8%) patients. No grade 4 or 5 TEAE or TEAE leading to discontinuation of T‐VEC was reported. Fourteen (77.8%) patients had TRAEs; the most frequent were pyrexia (44.4%), malaise (16.7%), chills, decreased appetite, pruritus, and skin ulcer (11.1% each).

**TABLE 2:** Patient incidence of AEs (safety analysis set; *n* = 18)

	T‐VEC (*n* = 18)			
	Any grade TEAE	Grade 3 TEAE	TRAE	SAE^a^
Any event	17 (94.4)	5 (27.8)	14 (77.8)	6 (33.3)
Pyrexia	9 (50.0)		8 (44.4)	
Nasopharyngitis	5 (27.8)			
Malaise	4 (22.2)		3 (16.7)	2 (11.1)
Arthralgia	2 (11.1)		1 (5.6)	
Chills	2 (11.1)		2 (11.1)	
Decreased appetite	2 (11.1)		2 (11.1)	1 (5.6)
Fatigue	2 (11.1)		1 (5.6)	
Nausea	2 (11.1)			
Pruritus	2 (11.1)		2 (11.1)	
Skin ulcer	2 (11.1)		2 (11.1)	
Acne	1 (5.6)			
Alanine aminotransferase increased	1 (5.6)		1 (5.6)	
Alopecia areata	1 (5.6)		1 (5.6)	
Arthritis	1 (5.6)		1 (5.6)	
Aspartate aminotransferase increased	1 (5.6)		1 (5.6)	
Benign prostatic hyperplasia	1 (5.6)		1 (5.6)	
Diarrhea	1 (5.6)		1 (5.6)	
Enteritis infectious	1 (5.6)	1 (5.6)		1 (5.6)
Epiglottitis	1 (5.6)	1 (5.6)		1 (5.6)
Forearm fracture	1 (5.6)	1 (5.6)		1 (5.6)
Hematuria	1 (5.6)		1 (5.6)	
Hyperglycemia	1 (5.6)	1 (5.6)		
Injection‐site reaction	1 (5.6)		1 (5.6)	
Jaundice cholestatic	1 (5.6)	1 (5.6)		1 (5.6)
Laryngeal pain	1 (5.6)		1 (5.6)	
Pain	1 (5.6)		1 (5.6)	
Pneumonia	1 (5.6)	1 (5.6)		1 (5.6)
Pollakiuria	1 (5.6)		1 (5.6)	
Rash	1 (5.6)		1 (5.6)	
Syncope	1 (5.6)	1 (5.6)		
Vitiligo	1 (5.6)		1 (5.6)	
Malignant melanoma				1 (5.6)

*Note*: Data are presented as number (%) of patients. Events of any grade that occurred in ≥10% of patients are shown. All grade 3 TEAEs, TRAEs, and SAEs are shown. The safety analysis set included all enrolled patients who received ≥1 dose of T‐VEC. TEAEs were defined as AEs occurring from day 1 to 30 days after the last treatment.

Abbreviations: AE, adverse event; *n*, number of patients in the safety analysis set; SAE, serious adverse event; TEAE, treatment‐emergent adverse event; TRAE, treatment‐related adverse event; T‐VEC, talimogene laherparepvec.

^a^ SAEs were defined as any AE with an onset date from the first dose to 90 days after the last dose of T‐VEC or 30 days following cessation of treatment if the patient initiated new anticancer therapy, whichever was earlier. SAEs are not a subset of any grade AEs as they could include events outside the TEAE period (after 30 days per definition). This study considered serious disease‐related AEs separately from SAEs.

Six (33.3%) patients had ≥1 SAE (Table 2). The only SAE that occurred in >1 patient was grade 2 malaise; one of these events was considered related to T‐VEC. One additional SAE of grade 2 decreased appetite was also considered related to T‐VEC and coincided with the SAE of malaise in the same patient. In addition to these two SAEs of malaise and decreased appetite (with clinical course described below), the remaining seven SAEs reported in five patients were not considered related to T‐VEC.

An 80‐year‐old female patient experienced SAEs of grade 2 malaise and grade 2 decreased appetite, both attributed to T‐VEC. She had received the first dose of T‐VEC 2 months prior to the onset of the SAEs and was hospitalized for 10 days, starting 2 days after her sixth dose of T‐VEC. She improved with supportive care and continued with T‐VEC dosing for another 2 months before ending treatment due to requirement for alternative therapy.

### 3.4 Response to therapy

**FIGURE 2:**
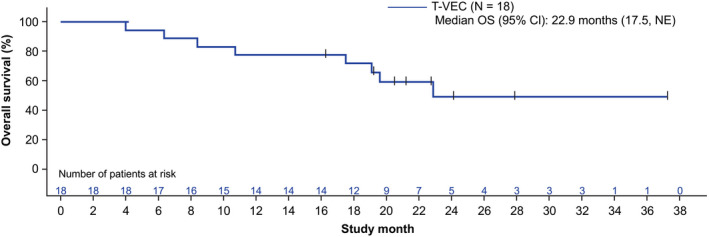
Overall survival (safety analysis set). CI, confidence interval; NE, not estimable; OS, overall survival; T‐VEC, talimogene laherparepvec. Censor indicated by vertical bar |. OS was defined as the interval from the first dose to the event of death from any cause; otherwise, OS was censored at the date the patient was last known to be alive. The safety analysis set included all patients who received ≥1 dose of T‐VEC

**FIGURE 3:**
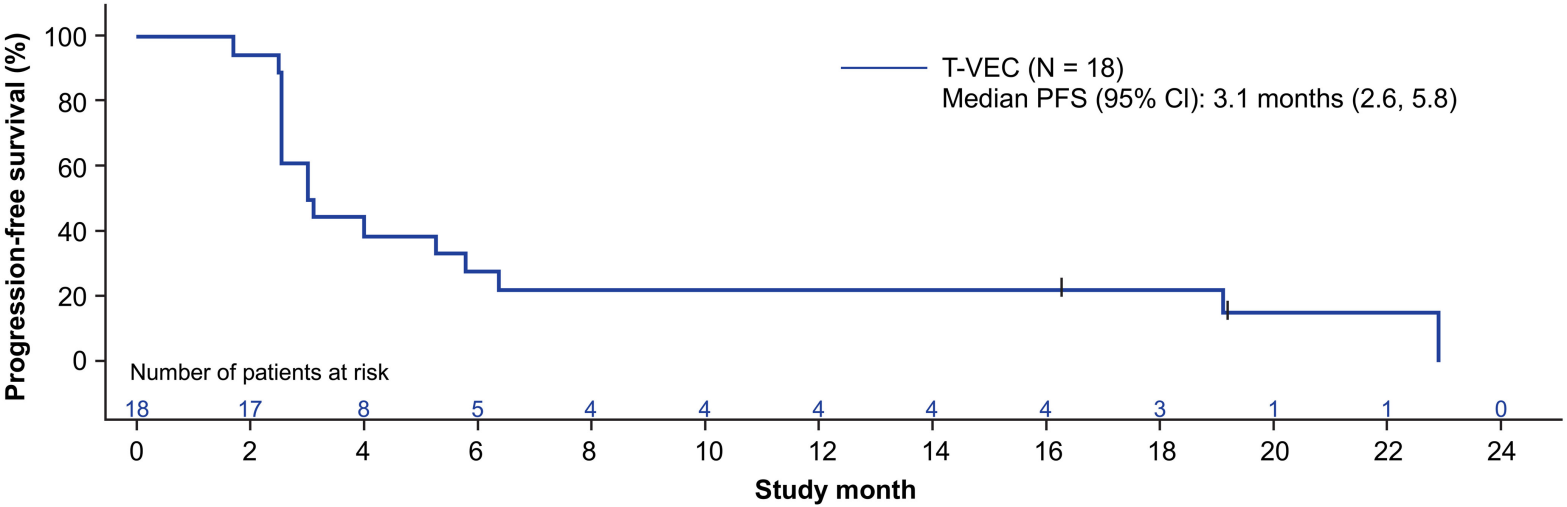
Progression‐free survival (safety analysis set). CI, confidence interval; PFS, progression‐free survival; T‐VEC, talimogene laherparepvec; WHO, World Health Organization. Censor indicated by vertical bar |. PFS per modified WHO response criteria was defined as the interval from the first dose to the earlier of disease progression per modified WHO response criteria or death from any cause; otherwise, PFS was censored at the last evaluable tumor assessment. The safety analysis set included all patients who received ≥1 dose of T‐VEC

Two (11.1%) patients showed a durable PR ≥6 months (Table 3). Both patients had stage IVM1b‐c disease and had received ≥2 prior lines of therapy that included nivolumab and ipilimumab treatment. One patient with a durable response had ALM and the other had NM. The DRR was 11.1% (95% CI: 1.4, 34.7), the ORR was 11.1% (95% CI: 1.4, 34.7), and the median TTR and DOR were NE. The median time to death was 22.9 (95% CI: 17.5, NE) months (Figure 2). Four (22.2%) patients had best overall response of stable disease (Table S3). Sixteen (88.9%) patients had disease progression. The median time to disease progression or death was 3.1 (95% CI: 2.6, 5.8) months (Figure 3).

**TABLE 3:** Response to therapy (safety analysis set; *n* = 18)

	T‐VEC (*n* = 18)
DRR, % (95% CI^a^)	11.1 (1.4, 34.7)
ORR, % (95% CI^a^)	11.1 (1.4, 34.7)
TTR (KM) (responders only), months	
Mean (SE)	7.63 (0.61)
Median (95% CI)	NE (8.08, NE)
PFS (KM), months	
Median (95% CI)	3.07 (2.56, 5.78)
OS (KM), months	
Median (95% CI)	22.87 (17.51, NE)

*Note*: DRR was defined as the rate of objective response (CR or PR) lasting continuously for ≥6 months and starting any time within 12 months of initiating therapy. ORR was defined as the incidence of an objective response of CR or PR per modified WHO response criteria among the set of patients analyzed.

Abbreviations: CI, confidence interval; CR, complete response; DRR, durable response rate; KM, Kaplan–Meier; *n*, number of patients in the safety analysis set; NE, not estimable; ORR, overall response rate; OS, overall survival; PFS, progression‐free survival; PR, partial response; SE, standard error; TTR, time to response; T‐VEC, talimogene laherparepvec; WHO, World Health Organization.

^a^ Binomial proportion with exact 95% CI.

### 3.5 Unintended exposure to T‐VEC

No cases of unintended exposure to T‐VEC were reported in patients' close contacts or healthcare providers. The one case of herpetic lesion reported in this study, which was sampled from a close contact, was confirmed to be negative for T‐VEC DNA.

## 4 DISCUSSION

Talimogene laherparepvec was well tolerated and showed clinical activity in Japanese patients with unresectable stage IIIB–IV malignant melanoma; no DLTs were reported and the DRR was 11.1%.

Overall, the safety and efficacy findings in this study are consistent with the results of the OPTiM trial, which compared intralesional T‐VEC with subcutaneous GM‐CSF in non‐Asian patients with unresectable stage IIIB–IV melanoma.^27^ OPTiM was the first randomized controlled phase III study to demonstrate a therapeutic benefit of an oncolytic immunotherapy in patients with melanoma; T‐VEC monotherapy demonstrated a tolerable safety profile and resulted in a DRR of 16.3% and a CR rate of 10.8%. In OPTiM, the most common AEs in T‐VEC–treated patients were fatigue, chills, pyrexia, nausea, influenza‐like illness, and injection‐site pain, and most AEs were mild to moderate. Consistent with these data, in the current study, most TEAEs were grade 1 or 2, and the most frequently reported TEAEs were pyrexia (50.0%), nasopharyngitis (27.8%), and malaise (22.2%).

Melanoma subtypes differ in risk factors, epidemiology, and tumor biology, therefore raising the question whether subtypes respond differently to treatment.^1^, ^3^, ^4^ ALM and MM are rare melanoma subtypes; however, in individuals with darker pigmentation in whom the overall incidence of melanoma is low, these subtypes are relatively more common. Efficacy in ALM and MM is not reported separately in most clinical trials that enroll patients with these subtypes. Recent data according to Hospital‐Based Cancer Registries and nationwide statistical surveys in Japan showed that the ALM subtype was observed in 41%, NM in 20%, SSM in 19%, and LMM in 7% of cases.^3^ In the current study, SSM and NM were the predominant subtypes followed by three cases of ALM. The durable PR observed in one patient with ALM supports another published case report and an anecdotal case treated at the European Institute of Oncology that shows T‐VEC activity in this subtype.^29^, ^30^ Notably, the prevalence of subtypes in this study is not typical of the Japanese population. The relatively similar prevalence of subtypes in this study compared with studies in Western countries suggests that the results observed may be comparable with the results observed in Western populations.

Due to the nature of the study, the small sample size limited the analysis of efficacy within the patient subgroups. Finally, this study included only Japanese patients; therefore, the results may not be generalizable to other Asian populations.

The development of oncolytic viral therapies such as T‐VEC opens novel avenues for the management of unresectable melanoma. This study established the safety, tolerability, and efficacy of T‐VEC in a small number of Japanese patients with advanced melanoma, including rare subtypes. The DRR was 11.1% (95% CI: 1.4, 34.7) and the overall safety profile was consistent with patients' underlying disease and the known safety profile of T‐VEC. These data support further investigation of T‐VEC in Asian patients with advanced melanoma.

## AUTHOR CONTRIBUTIONS

Naoya Yamazaki: Conceptualization, Investigation, Supervision, Writing – review & editing. Taiki Isei: Investigation, Writing – review & editing. Yoshio Kiyohara: Investigation, Writing – review & editing. Hiroshi Koga: Investigation, Writing – review & editing. Takashi Kojima: Conceptualization, Investigation, Writing – review & editing. Tatsuya Takenouchi: Investigation, Writing – review & editing. Kenji Yokota: Investigation, Writing – review & editing. Kenjiro Namikawa: Investigation, Writing – review & editing. Min Yi: Data curation, Formal analysis, Writing – review & editing. Alissa Keegan: Project administration, Supervision, Writing – original draft, Writing – review & editing. Satoshi Fukushima: Investigation, Writing – review & editing.

## CONFLICT OF INTEREST

Naoya Yamazaki received fees for an advisory role from Ono Pharmaceutical, Chugai Pharma, and MSD; fees for speakers' bureau from Ono Pharmaceutical, Bristol‐Myers Squibb Japan, Novartis, and MSD; and research funding from Ono Pharmaceutical, Bristol‐Myers Squibb Japan, Novartis, Astellas, Amgen BioPharma, Merck BioPharma, and Takara Bio. Taiki Isei received honoraria from Ono Pharmaceutical, Bristol‐Myers Squibb, Novartis Pharma, and MSD and fees for consulting or advisory role from Ono Pharmaceutical, Bristol‐Myers Squibb, and Novartis Pharma. Yoshio Kiyohara has no conflict of interest. Hiroshi Koga has no conflict of interest. Takashi Kojima reports grants and personal fees from Ono Pharmaceutical, grants and personal fees from MSD, grants from Astellas Amgen BioPharma, grants from Taiho Pharmaceutical, grants from Shionogi, personal fees from Oncolys BioPharma, personal fees from Astellas Pharma, personal fees from Bristol‐Myers Squibb, and personal fees from Merk. Tatsuya Takenouchi received fees for speakers' bureau from Ono Pharmaceutical, Novartis Pharma, MSD, and Bristol‐Myers Squibb. Kenji Yokota has no conflict of interest. Kenjiro Namikawa received honoraria from Ono Pharmaceutical, Novartis Pharma, MSD, and Bristol‐Myers Squibb, outside the submitted work. Min Yi has no conflict of interest. Alissa Keegan is an employee and stockholder of Amgen Inc. Satoshi Fukushima has no conflict of interest.

The study was designed under the responsibility of Amgen Inc., in conjunction with the steering committee. The study was funded by Amgen Inc. Talimogene laherparepvec was donated/provided by Amgen Inc. Amgen Inc. collected and analyzed the data and contributed to the interpretation of the study. All authors had full access to all the data in the study and had final responsibility for the decision to submit for publication.

## ETHICS STATEMENT

This study (ClinicalTrials.gov identifier: NCT03064763) was conducted in accordance with the Declaration of Helsinki and International Council for Harmonization Good Clinical Practice guidelines. Approval was obtained from the appropriate ethics committees or institutional review boards and all patients provided written informed consent before study entry.

## Supporting information


Figure S1
Table S1
Table S2
Table S3

## Data Availability

Qualified researchers may request data from Amgen clinical studies. Complete details are available at: http://www.amgen.com/datasharing
