# Supplementary material for: A phase I study of the safety and efficacy of talimogene laherparepvec in Japanese patients with advanced melanoma
Source: Cancer Sci. 2022 Jun 30;113(8):2798–806. doi: 10.1111/cas.15450 (PMC9357627; doi:10.1111/cas.15450)
Supplement: Supplementary file 1 — Figure S1 Table S1 Table S2 Table S3 [file CAS-113-2798-s001.docx]

**Title: A phase I study of the safety and efficacy of talimogene laherparepvec in Japanese patients with advanced melanoma**

**SUPPLEMENTAL MATERIAL**

**FIGURE S1.** Study design and treatment schema.

**
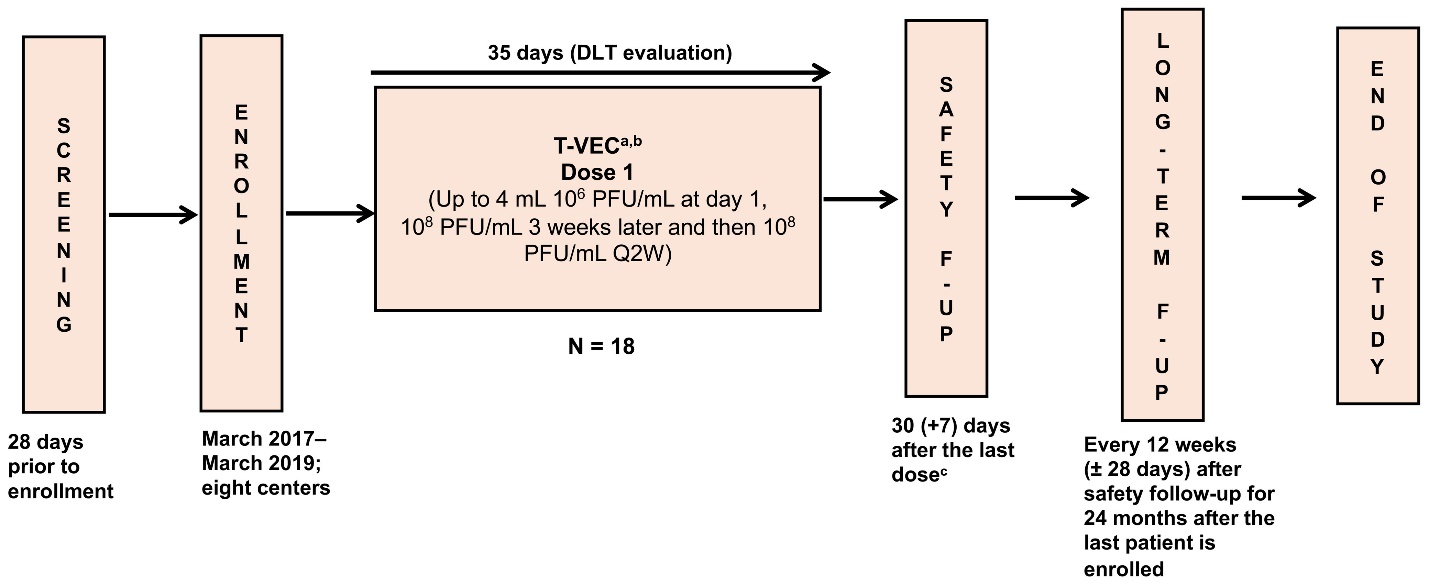
**

CR, complete response; DLT, dose-limiting toxicity; Q2W, every 2 weeks; T-VEC, talimogene laherparepvec; WHO, World Health Organization.

^a^Up to approximately 18 patients were enrolled in the study. Patients were included in the evaluation for both safety and efficacy. The DLT evaluation period was 35 days from the initial administration of T-VEC. Initially, six DLT-evaluable patients were enrolled and treated with dose 1. Upon demonstration of safety based on DLT rules, additional 12 patients were enrolled and treated with dose 1 to obtain additional safety data. However, if dose 1 was declared unsafe based on DLT rules, then additional six DLT-evaluable patients were to be enrolled at dose -1.

^b^Treatment was continued until patient experienced a DLT (during the DLT evaluation period), patient achieved a CR, had no injectable lesions, had clinically relevant (resulting in clinical deterioration or requiring change of therapy) disease progression beyond 24 weeks of treatment per modified WHO response criteria, had a safety concern, or had a maximum treatment duration of 48 months, whichever occurred first.

^c^All patients were to complete a safety follow-up visit 30 (+ 7) days after the last dose of T‑VEC. Patients were followed for survival, subsequent anticancer therapies, and T-VEC–related adverse events every 12 weeks (± 28 days) for 24 months after the last patient was enrolled. For patients treated beyond 24 months after the last patient was enrolled, their final visit was to serve as the safety follow-up visit.

## TABLE S1. Disease status at screening and lesion injection details of T-VEC for Day 1

| **Patient ID** | **Stage at screening** | **Distant metastatic sites at screening** | **Number of lesions injected** | **Lesion type injected** | **Body site location injected** | **Best overall response** |
| --- | --- | --- | --- | --- | --- | --- |
| 1 | IVM1c | Lung and inguinal lymph nodes | 6 | Subcutaneous | Inguinal and leg | PD |
| 2 | IVM1b | Lung | 20 | Cutaneous | Leg | PR |
| 3 | III | N/A | 1 | Subcutaneous | Inguinal | Missing |
| 4 | IIIC | N/A | 5 | Subcutaneous | Leg | Stable disease |
| 5 | IVM1a | Neck lymph nodes | 1 | Nodal | Neck | PD |
| 6 | IVM1a | Left lower extremity | 8 | Cutaneous | Leg | Stable disease |
| 7 | IVM1a | Abdominal and pelvic lymph nodes | 2 | Subcutaneous | Leg | PD |
| 8 | IVM1c | Neck and axillary lymph nodes | 1 | Nodal | Axillary lymph node | PD |
| 9 | IVM1a | Extremities | 5 | Subcutaneous | Neck, back, trunk, and gluteal | PD |
| 10 | IVM1c | Supraclavicular lymph node | 1 | Nodal | Supraclavicular lymph node | PD |
| 11 | IIIC | N/A | 2 | Subcutaneous | Leg | Stable disease |
| 12 | IVM1c | Neck lymph nodes | 1 | Subcutaneous | Face | Stable disease |
| 13 | IVM1a | Right extremity | 5 | Subcutaneous | Leg | PD |
| 14 | IIIB | N/A | 1 | Subcutaneous | Leg | PD |
| 15 | IVM1a^a^ | Lung^a^ | 1 | Subcutaneous | Leg | Missing |
| 16 | IVM1c | Pelvic lymph nodes | 1 | Cutaneous | Gluteal | PD |
| 17 | IVM1c | Subcutaneous and liver | 1 | Cutaneous | Leg | PR |
| 18 | IVM1b^b^ | Kidney^b^ | 1 | Subcutaneous | Back | PD |

N/A, not available; n, number of patients in the safety analysis set; PD, disease progression; PR, partial response; T-VEC, talimogene laherparepvec.

## ^a^Lung metastases were identified on screening scans, which would upgrade stage from IVM1a (investigator assigned) to M1b

## ^b^Kidney metastasis was identified on screening scans, which would upgrade from stage of IVM1b (investigator assigned) to M1c

## TABLE S2. Summary of prior anticancer therapy (all enrolled patients)

|  | **T-VEC** |
| --- | --- |
|  | **(n = 18)** |
| Number of prior lines of therapy |  |
| None | 2 (11.1) |
| 1 | 3 (16.7) |
| 2 | 7 (38.9) |
| 3 | 5 (27.8) |
| 4 | 0 (0.0) |
| > 4 | 1 (5.6) |
|  |  |
| Type of prior anticancer therapy (administered as either monotherapy or in combination) |  |
| Immunotherapy | 14 (77.8) |
| PD-1/PD-L1 | 11 (61.1) |
| CTLA-4 | 2 (11.1) |
| Interferon | 9 (50.0) |
| Other | 2 (11.1) |
| Chemotherapy | 2 (11.1) |
| Hormonal therapy | 1 (5.6) |
| Targeted biologics | 1 (5.6) |
| Targeted small molecules | 1 (5.6) |
| Not applicable | 4 (22.2) |
| Other | 1 (5.6) |
|  |  |
| Reason for stopping prior therapy |  |
| Disease progression | 15 (83.3) |
| Completed treatment course | 5 (27.8) |
| Clinical progression | 2 (11.1) |
| Patient’s decision | 2 (11.1) |
| Investigator decision | 1 (5.6) |
| Toxicity | 1 (5.6) |
| Complete response | 0 (0.0) |
| Other | 1 (5.6) |
| Unknown | 1 (5.6) |
|  |  |

*Note*: Data are presented as number (%) of patients.

The subcategories within each category are not mutually exclusive, except "Number of prior lines of therapy".

CTLA-4, cytotoxic T-lymphocyte-associated antigen 4; PD-1, programmed cell death protein 1; PD-L1, programmed death-ligand 1; T-VEC, talimogene laherparepvec.

## TABLE S3. Analysis of best overall response rate per modified WHO response criteria by investigator (safety analysis set; n = 18)

|  | **T-VEC** |
| --- | --- |
|  | **(n = 18)** |
|  |  |
| Response assessment based on investigator |  |
| Complete response (CR) | 0 (0.0) |
| Partial response (PR) | 2 (11.1) |
| Stable disease | 4 (22.2) |
| Disease progression | 10 (55.6) |
| Missing | 2 (11.1) |
|  |  |
| Overall response rate (CR/PR) | 2 (11.1) |
| 95% CI^a^ | 1.4, 34.7 |

*Note*: Data are presented as number (%) of patients.

CI, confidence interval; n, number of patients in the safety analysis set; T-VEC, talimogene laherparepvec; WHO, World Health Organization.

Overall response rate was defined as the incidence of an objective response of CR or PR per modified WHO response criteria among patients in the safety analysis set.

The safety analysis set included all enrolled patients who received ≥ 1 dose of T-VEC.

## ^a^Binomial proportion with exact 95% CI.
